# Intramedullary schwannoma of conus medullaris with syringomyelia: a case report and literature review

**DOI:** 10.3389/fonc.2024.1422958

**Published:** 2024-12-02

**Authors:** Hua Guo, Yao Wang, Liankun Wang, Dianhui Han, Xiangyi Meng, Qingchun Mu, Xiaofeng Chen

**Affiliations:** ^1^ Department of Neurosurgery, The First Affiliated Hospital of Harbin Medical University, Harbin, Heilongjiang, China; ^2^ Department of Neurosurgery, The Second Affiliated Hospital of Soochow University, Suzhou, Jiangsu, China; ^3^ Department of Neurology, Heilongjiang Province Hospital, Harbin, Heilongjiang, China

**Keywords:** intramedullary schwannoma, syringomyelia, conus medullaris, surgical treatment, case report

## Abstract

Intramedullary schwannomas in the conus medullaris are very rare and are usually not associated with syringomyelia. We report a unique case of intramedullary schwannoma in the conus medullaris with long-segment syringomyelia. The patient was a 60-year-old male, initially presenting with left dorsalgia, subsequently developing weakness in the right lower extremity. As the symptoms progressed, the patient exhibited ataxia in gait, accompanied by sphincter insufficiency and voiding dysfunction. Lumbar MRI revealed the presence of two tumors at the L3 and T11-L1 levels, accompanied by syringomyelia extending from T4 to T10. During surgery, it was determined that the tumor located at the T11-L1 vertebral level was intramedullary, whereas the tumor situated at the L3 level exhibited an extramedullary intradural configuration. Pathological examination conclusively identified both the intramedullary and extramedullary tumors as schwannomas. Although intramedullary schwannomas at the conus medullaris are very rare, schwannoma remains a diagnosis that cannot be ignored when facing patients with intramedullary tumors with syringomyelia. Intramedullary schwannoma can have a good neurological prognosis after surgical treatment.

## Introduction

Schwannomas are one of the most common primary spinal tumors. It usually originates from Schwann cells of the peripheral nervous system, and most of them are solitary and accompanied by a capsule. Based on the different positional relationships with the spinal dura mater, spinal schwannomas can be classified into three types: epidural (8-32%), intradural-extramedullary (1-19%), and intradural(49-83%) (3, 4). The vast majority of schwannomas are located in the intradural space. Intradural schwannomas can be categorized into extramedullary intradural and intramedullary(IM) schwannomas. IM schwannomas are considered rare neoplasms, comprising only 1.1% of spinal schwannomas and 0.3% of all IM tumors (7). The most common site of IM schwannomas is the cervical spine and the level of the conus medullaris are less common, and those with syringomyelia are even rarer (11). We describe the first-ever instance to our knowledge of IM schwannoma of conus medullaris with long segment syringomyelia.

## Case report

Anal sphincter weakness, urinary difficulties for two months, right lower limb weakness and walking instability for half a year, and left back pain for a year all are the symptoms which brought the 60-year-old male patient to the hospital. Neurological examination revealed sensory impairment below the inguinal plane, disappearance of abdominal wall reflex and cremasteric reflex. After admission, ultrasound examination showed that the residual urine volume in the bladder after urination was 530 ml. The patient did not have any previous major illness and had only taken oral pain medication in the past, this was his first visit to the hospital. Even with painkillers, his symptoms got worse. None of the patient’s family members had a history of schwannoma.

**Figure 1:**
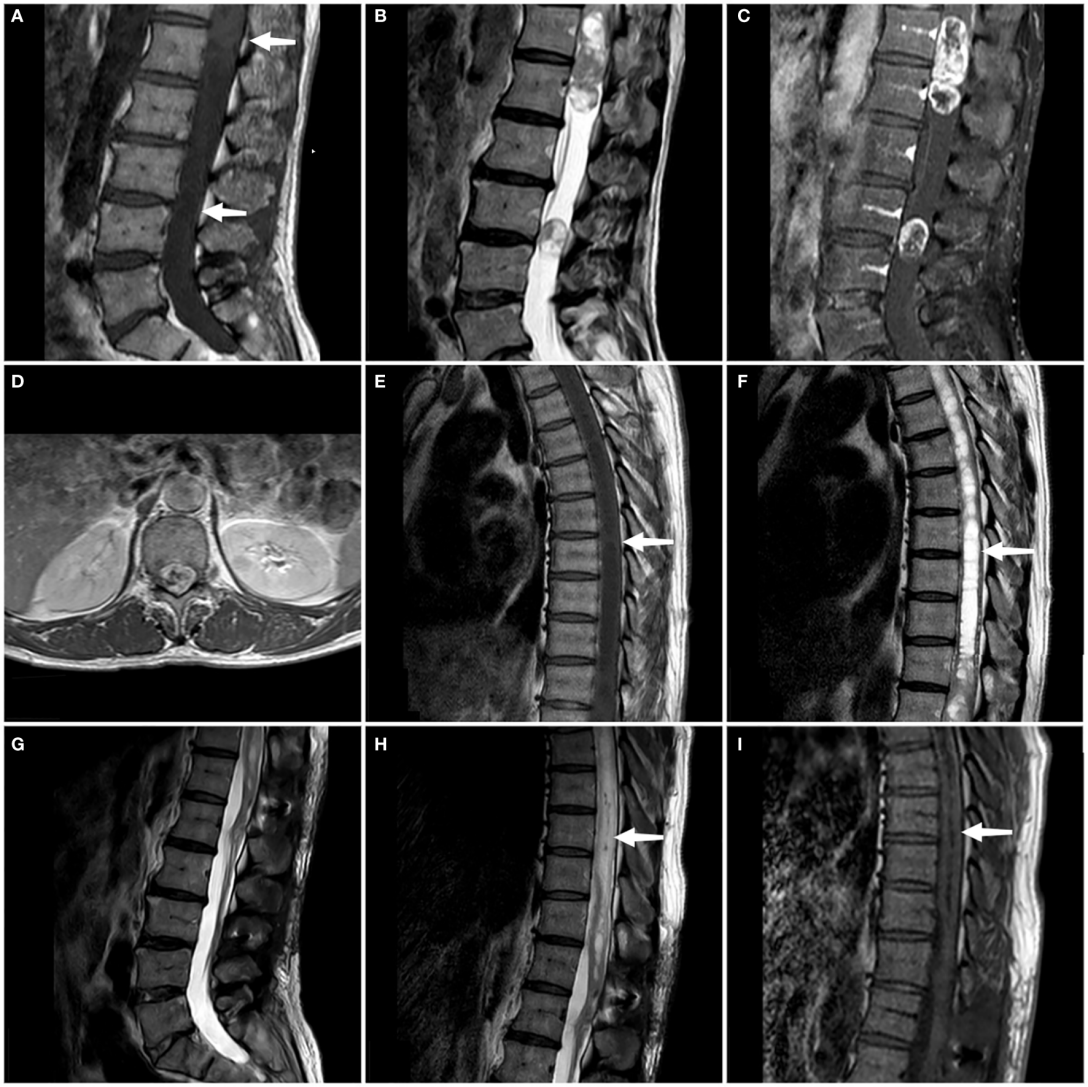
On the sagittal T1WI sequence, iso-signal occupying lesions (at the white arrow) are discernible separately within the T11-L1 segment and the L3 segment **(A)**. On the sagittal T2WI sequence, both tumors exhibit mixed signals **(B)**. Enhanced T1WI scans in both sagittal and axial planes reveal significant and heterogeneous enhancement of both lesions **(C, D)**. A persistent intramedullary abnormal signal at T4-T10 was seen above the lesion (at the white arrow), showing hypointense on the T1WI and hyperintense on the T2WI **(E, F)**. Follow-up MRI at 3 months confirmed complete tumor removal with no recurrence **(G)**. Sagittal T1- and T2-weighted thoracic MRI images reveal a marked reduction in syringomyelia compared to preoperative findings (white arrow) **(H, I)**.

We conducted a comprehensive MRI scan of the patient’s entire spine, which included T1-weighted imaging (T1WI), T2-weighted imaging (T2WI), and enhanced scanning following the injection of a contrast agent. The tumors showed mixed signals on MRI plain scan and heterogeneous enhancement on enhanced MRI.MRI examination showed two tumors at L3 level and T11-L1 level respectively, measuring 8 × 8 × 10 mm and 24 × 54 × 20 mm. At the same time, syringomyelia at T4-T10 level appeared above the tumor at T11-L1 level (**Figure 1**). Based on the patient’s clinical manifestations and MRI examination, the preoperative preliminary diagnosis was schwannoma or ependymoma. Other differential diagnoses included glioma, astrocytoma, calcified tuberculoma etc.

Subsequently, both tumors were removed simultaneously. The patient was placed in the prone position and two incisions were made at T12-L1 and L3 spinous processes. The skin and fascia were dissected layer by layer, and the muscles on both sides of the spinous process were separated and pulled. The bilateral laminae of T12, L1 and L3 were opened to expose the tumors, revealing that the tumor at the T11-L1 level was IM, while the tumor at L3 was extramedullary intradural.

**Figure 2:**
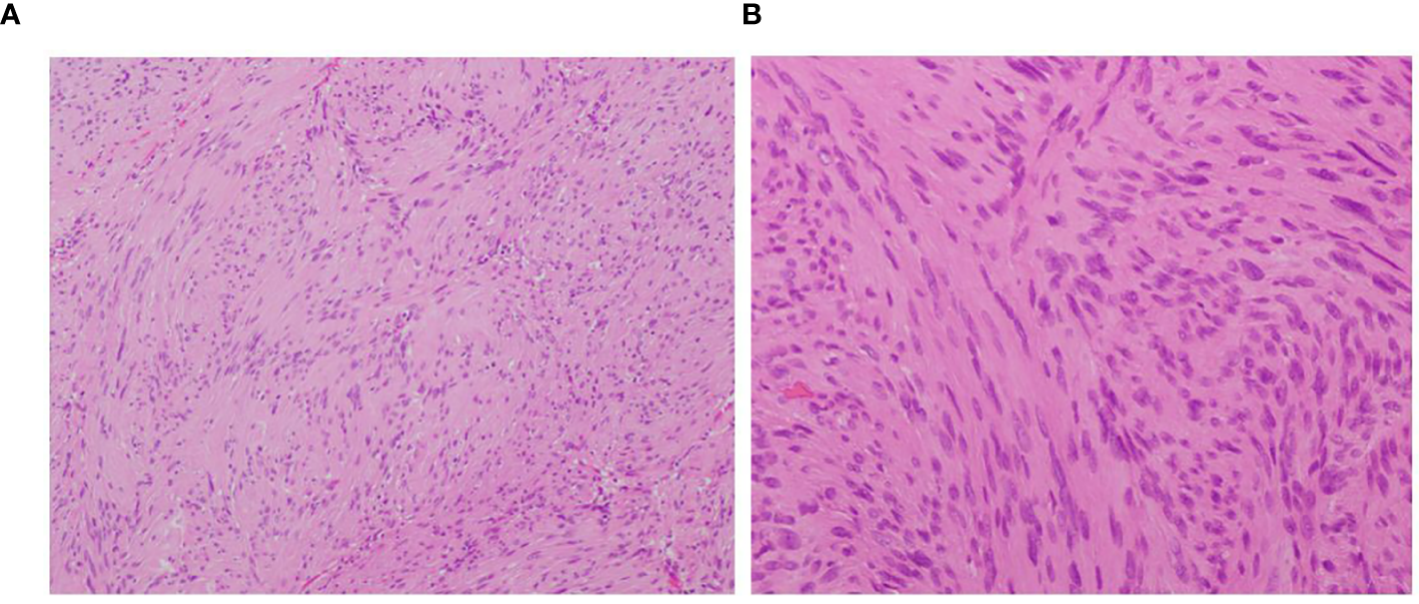
**(A)** observing under a microscope, the tumor shows cellular and hypocellular areas (H&E×200). **(B)** plumped spindle-shaped cells with palisading nuclei can be seen (H&E ×400).

Both tumors were completely resected under the microscope, the patient’s neurological symptoms improved after the operation. Pathological examination showed that both tumors were schwannomas (**Figure 2**).

**Figure 3:**
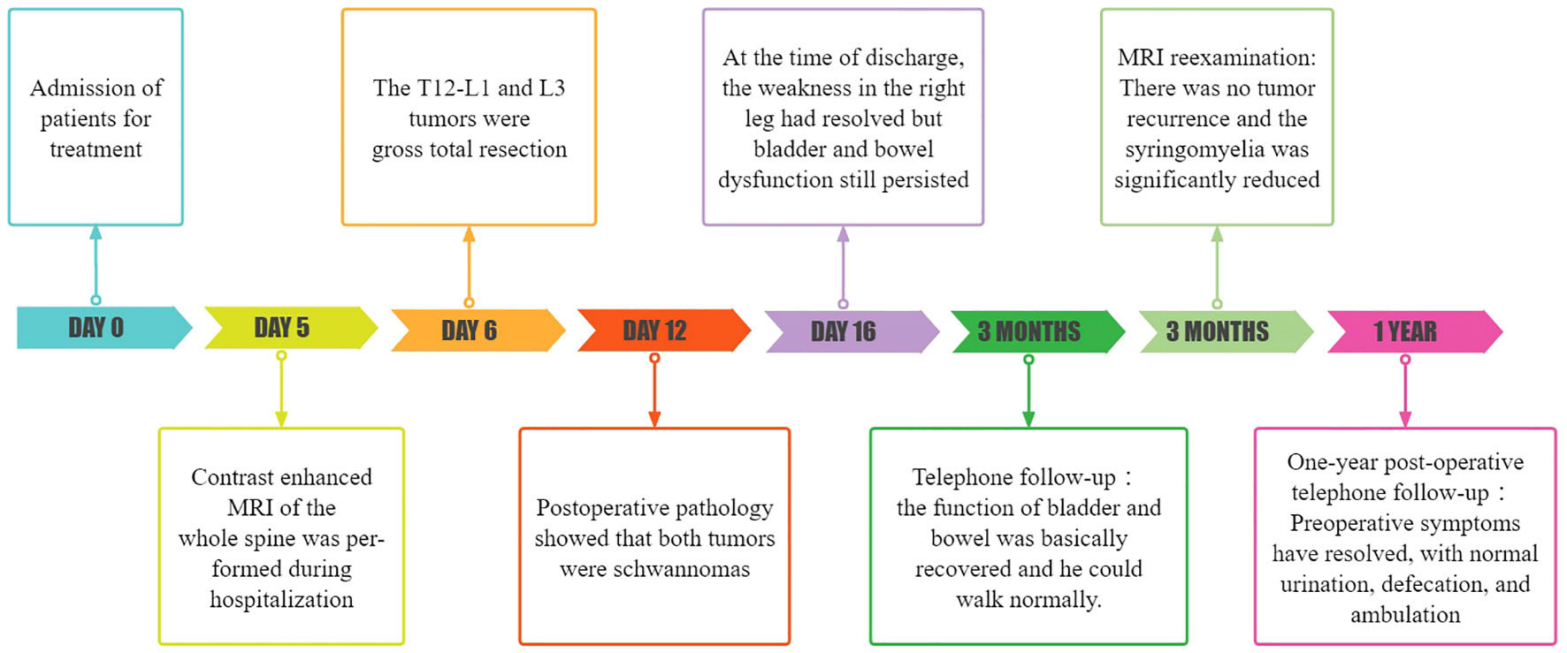
Treatment timeline of the patient.

At the time of discharge, the patient’s condition was significantly better than that on admission, the weakness of the right lower limb was significantly relieved, and he could basically walk normally. He still had weakness of the anal sphincter and difficulty in bowel and defecation. The lumbar incision healed well. Physical examination showed grade 5 muscle strength in the right lower extremity and Babinski signs were negative. Three months later, the patient’s bladder and stool function was significantly improved compared with that before surgery, and he could walk completely independently. MRI reexamination showed that the tumor was completely removed and no recurrence was observed, and syringomyelia significantly shrank (**Figure 1**). During the one-year post-operative telephone follow-up, the patient is currently able to walk normally, with urinary and bowel functions having largely returned to normal, and the preoperative symptoms have essentially dissipated.The treatment timeline is shown in **Figure 3**.

## Discussion

IM schwannomas are very rare, pathologist James Kernohan first reported IM schwannoma in 1931 (17). The last possible literature review was done in 2021 by V. M. Swiatek et al. that listed 166 IM schwannoma instances that were documented at the time (11). The cervical spine accounted for 63% of recorded cases of IM schwannoma, with the thoracic spine of the spinal cord coming in second at 26% and the lumbar spine at 11% (21). We conducted a systematic review of the literature in PubMed up to January 1, 2023 using the keywords “intramedullary” and “schwannoma” to retrieve all relevant studies and case reports of IM schwannoma. Inclusion criteria were as follows (1): at least one histologically confirmed IM schwannoma was reported (2), the tumor was located at the site of the conus medullaris, and (3) clinical information on the patient was available. We did not include the cases with neurofibromatosis. Only 15 cases—not including this one—of IM schwannoma in the spinal cord’s conus have ever been documented in the literature (1, 2, 5, 6, 8–10, 12, 13, 15, 16, 18–20, 22) (**Table 1**).

**Table 1:** Summary of conus intramedullary schwannomas (in non-neurofibromatosis patients) reported to date.

Author reference	S. No.	Age (years)	Sex	Date	Tumor level	Symptom; duration	SM	MRI	Clinico-radiological differentials	Treatment	Resection	Follow up details available
Our case	1	60	M	2023	T11-L1	Back pain; 1 year	Yes	Inhomogeneous enhancement	Ependymoma Astrocytoma	T11-L1laminectomy	GTR	3 months, recovering well
Rahul Varshney et al. (2)	2	70	M	2020	T11-L2	Paraparesis, sphincter dysfunction; 2.5 year	No	Inhomogeneous enhancement	Ependymoma Astrocytoma	D12-L1 laminectomy	GTR	3 months, recovering well
Ritika Singh et al. (6)	3	27	F	2018	Conus	Lower backache; 1 year	No	Heterogenous rim enhancement	Ependymoma Astrocytoma	T12-L1 laminectomy	STR	1 year, recovering well
Karatey et al. (9)	4	30	F	2017	T12–L1	Back pain, walking difficulty; 2 months	Yes	Homogeneous	Astrocytoma	T12 laminectomy	GTR	N/A
								enhancement				
Jagannatha et al. (12)	5	11	M	2016	T11–12	Weakness of both legs; 1 year	Yes	Homogeneous	Ependymoma Astrocytoma Calcified tuberculoma	T10–D12 laminotomy	GTR	6 months, recovering well
								enhancement				
Yang et al. (14)	6	35	M	2014	T11–L2	Lower back pain, weakness in the left leg; 2 years	Yes	Inhomogeneous enhancement	Ependymoma	T11–12 laminectomy	GTR	9 months, full recovery
Kumar et al. (16)	7	40	F	2014	Conus	Back pain, walking difficulty; 1.5–2 years	N/A	Homogeneous	N/A	T10–L1 laminectomy	GTR	1 month, partial recovery
								enhancement				
Canbay et al. (18)	8	49	F	2011	Conus	Lower back pain; 2 years	No	Inhomogeneous enhancement	N/A	T12–L1 laminectomy	GTR	N/A
Ohtonari et al. (19)	9	29	M	2009	Conus	Bladder dysfunction, paresthesia; 8 months	No	Homogeneous, well-enhanced,cystic lesion	N/A	T12–L1 laminectomy	STR	Patient being closely monitored for recurrence
M N Saiful Azl et al. (20)	10	54	M	2007	Conus	Low back pain; 2 years	No	Heterogenously enhanced	Ependymoma	laminectomy	GTR	N/A
Kahilogullari et al. (1)	11	338	F	2005	Conus	Waist and legs pain; 7 months	No	Homogeneous, well-enhanced	N/A	T12–L1 laminectomy	STR	Partial recovery postop
O’Brien et al. (5)	12	448	M	2003	T11-L1	Weakness in the legs; 6 month**s**	No	Equal signal on T2	N/A	T11–L1 laminectomy	STR	6 month**s**, no recurrence
Lesoin et al. (8)	13	N/A	M	1983	Conus	Weakness in the legs; 5 years	N/A	N/A	N/A	N/A	GTR	Slight weakness in left leg at 11 months
Schmitt (10)	14	N/A	M	1975	Conus	Paresthesia; 1.5 months	N/A	N/A	N/A	Autopsy finding	N/A	N/A
Guidetti (13)	15	N/A	N/A	11965	Conus	N/A	N/A	N/A	N/A	N/A	GTR	N/A
McCormik (15)	16	N/A	M	1964	L2	1.5 months	N/A	N/A	N/A	Autopsy finding	N/A	N/A

N/A, not applicable.

By reviewing 16 cases with IM schwannomas in the conus of the spinal cord including our case, the age range was found to be 11–70 years old, with an average age of 40.1 ± 16.2 years. In a 2:1 ratio, men are more affected than women, which is consistent with the previous literature (11). The mean interval between the symptom onset and diagnosis was 16.9 months. This may be because IM schwannoma often grows slowly and infrequently exhibits clinical signs in its early stages. Among the 16 cases, the main symptom of about 57% was low back pain, 43% had movement disorders, 21% had paresthesia, 14% had sphincter dysfunction, and 1 patient had hypolibido or sexual dysfunction. Depending on where the tumor is located, the symptomatology of schwannomas might include neurological impairments as well as signs and symptoms (6). Prior research focusing on the clinical characteristics and surgical outcomes of patients with IM schwannomas has consistently demonstrated that pain or sensory disturbance constitutes the most prevalent initial symptom, followed by motor dysfunction, with sphincter dysfunction ultimately manifesting in the late stages (14).

Since there are no Schwann cells in the central nervous system’s white matter, the pathophysiology of IM schwannomas is up for discussion (23). Intramural Schwann cells arising from the embryonal neural tube; Schwann cells transforming from neuroectoderm into pial cells; Schwann cells migrating into the cord in response to cord trauma; Schwann cells proliferating in the spinal artery nerve fibers; Schwann cells extending into cord from the region where spinal nerve roots enter pia mater are some of the theories regarding the origin of IM schwannomas (5, 24, 25). The Neurospinal Society of Japan conducted a nationwide analysis on IM Schwannoma of the spinal cord (26). Two primary categories of tumor location and extension can be distinguished: intramedullary to extramedullary extension and fully intramedullary extension. Each type may have a distinct developing pathway in light of these mechanisms and the location of the tumor. The shape of the tumor may depend on where in the spinal cord the aforementioned mechanisms take place. For instance, it is believed that tumors arising in more central parts of the spinal cord have intramedullary lesions, whereas tumors arising at the surface of the spinal cord have exophytic lesions.

Preoperative diagnosis might be challenging. MRI is the modality of choice for diagnosing IM schwannomas. On T1-weighted images, these IM schwannomas often show low to intermediate signal strength. They may be heterogeneous on T2-weighted imaging, showing collagen deposition, hemorrhage, and focal areas of hyper- and hypo-intensity (18). Strong contrast enhancement is typically seen in schwannomas, most likely as a result of open-gap junctions, which are short, straight, patent, and readily communicate with a sizable extracellular space (9). Of the 15 cases we reviewed, MRI data of 11 patients could be obtained, and the other 4 patients only had the relevant description of myelography. Uniform or nodular strong contrast enhancement was seen in all but one case (no T1 enhanced scan was performed), which may be due to the abundant blood supply of the schwannoma. Of them, syringomyelia was present in just 3 cases, whereas 3 cases had uneven enhancement and 7 cases had uniform enhancement. In our case, both lesions showed isointensity on sagittal T1-weighted images, with a long strip of hypointense shadow above the lesion at T11-L1. A heterogeneously low-signal lesion at T11-L1 with a noticeable high signal extending upward is visible on the sagittal T2-weighted MRI, which is consistent with syringomyelia. Both lesions showed significant uneven enhancement and well-delineated masses on coronal enhanced T1-weighted images. Differential diagnosis typically encompasses all IM lesions with contrast enhancement, such as ependymomas, astrocytomas, hemangioblastomas, and metastatic tumors, which generally exhibit unclear tumor boundaries and are associated with spinal cord edema and tumor cysts (27). While a well-defined enhancement pattern can serve as a distinguishing feature between IM schwannomas and other IM tumors, our review identified that 27% (3/11) of the cases did not conform to this pattern, manifesting as heterogeneous or rim enhancement. Peritumoral edema or tumor cysts are also prevalent in IM schwannomas, as observed in our cases. In conclusion, it is challenging to establish a definitive preoperative diagnosis of IM schwannoma solely based on MRI findings.

After analyzing preoperative MRI characteristics of IM schwannomas that had previously been documented in the literature, Ho et al. (7) came to the conclusion that the lack of syringomyelia was a diagnostic MRI sign of IM schwannomas. However, some cases with IM schwannomas also have syringomyelia associated with them. Nine of the twenty instances with IM schwannoma reported by Yang et al. (22) had syringomyelia. The study conducted by V. M. Swiatek et al. (11) reported that 20.9% of the 165 IM schwannomas patients were found to have association with syringomyelia. In our case, the MRI showed very long segment syringomyelia at the level of T4-T10. The review of 16 cases, including the present one, resulted in the exclusion of 2 cases due to missing imaging data and the identification of 4 cases associated with syringomyelia (9, 12, 22). We speculate that a tumor that obstructed the flow of cerebrospinal fluid in the central canal produced the syringomyelia. As a result, our conclusion is that the absence of syringomyelia, although prevalent, lacks specificity. For tumors such as IM ependymoma, astrocytoma, and hemangioblastoma, which require differentiation from IM schwannoma, the presence of syringomyelia also lacks specificity. Notably, ependymoma, due to its location at the center of the spinal cord, is often accompanied by syringomyelia (28). Therefore, we propose that the absence of syringomyelia may have a certain role in the diagnosis of IM schwannoma, albeit lacking specificity. Pathological diagnosis constitutes an indispensable component in confirming the diagnosis of schwannomas. Histopathological examination typically reveals the tumor tissue arranged in alternating hypercellular and hypocellular areas (Antoni A and B), with tumor cells dispersed within a loose, mucinous stroma. These cells display an oval to spindle shape and form palisading patterns (**Figure 2**). Immunohistochemical staining often demonstrates positive expression for S-100 protein and Leu-7 (4).

As schwannoma is benign, gross total resection (GTR) is the preferred treatment for IM schwannoma. Nonetheless, not every patient may be able to achieve GTR. A well-defined tumor-spinal cord anatomical plane is critical for obtaining GTR; therefore, an ill-defined anatomical plane may mean that the tumor is difficult to achieve GTR even if it is benign. STR may also be necessary to prevent deterioration of neurological function if the lesion is adherent to the neural tissue (21). Furthermore, the residual tumor may be removed with a second surgery (29). After reporting 20 cases with IM schwannomas, 16 of which had GTR and 4 of which had subtotal resection (STR), Yang et al. (22) came to the conclusion that GTR or STR could result in a favorable clinical outcome. By reviewing reported 14 IM schwannoma cases (2 cases were excluded because surgical information was not available), GTR was achieved in 79% (11/14) of patients. In the study conducted by Yang et al. (14), the syrinx shrank in 77.8% (7/9) of patients with IM schwannoma accompanied by syringomyelia who underwent tumor resection only, with no syrinx enlargement observed. In our case, the shrinkage of syringomyelia was also observed during the radiological reassessment conducted three months post-surgery. Therefore, for syringomyelia secondary to IM schwannoma, additional drainage of the syrinx may not be necessary, as the syrinx may collapse following tumor resection (28). Adjuvant treatment modalities, such as radiotherapy and chemotherapy, have failed to exhibit favorable therapeutic outcomes in the context of IM spinal cord schwannomas. Conversely, adjuvant radiotherapy and chemotherapy may be utilized in the management of partially resected or recurrent IM spinal cord tumors, encompassing gliomas and ependymomas.Research is ongoing to develop novel therapeutic strategies for these patients, including targeted drug delivery and nanomedicine technologies (30).

Recurrence of IM schwannoma tumors is rare, even in cases with STR (14). M. Swiatek et al. (11) reported 165 patients with IM schwannomas with a mean follow-up of 34 months, and tumor recurrence was observed in only 4% of cases. Of the 15 cases of conical IM schwannomas we collected, follow-up information was not available in 6 cases. Tumor recurrence was not observed in the remaining 9 cases, even though 4 of them only achieved STR. During the follow-up period with an average duration of 6 months, almost all cases exhibited improved neurological function post-operatively, including 4 patients who underwent STR. In our case, the tumor achieved GTR because both tumors had clear boundaries with the surrounding tissue. Three months following surgery, a follow-up MRI revealed no signs of tumor recurrence and a considerable reduction in the edema surrounding the tumor. In conclusion, our analysis suggests that the prompt surgical management of symptomatic IM schwannomas, irrespective of the surgical approach employed (either GTR or STR), holds the potential to elicit substantial improvements in patient prognosis.

## Conclusion

In conclusion, although IM schwannomas in the conus medullaris are rare tumors, they should still be an option when diagnosing intramedullary tumors, especially with syringomyelia. As a benign tumor, intramedullary schwannoma can achieve good neurological outcome after surgical treatment.

## Data Availability

The datasets presented in this article are not readily available because of ethical and privacy restrictions. Requests to access the datasets should be directed to the corresponding authors.
